# Supplementary material for: MetaRibo-Seq measures translation in microbiomes
Source: Nat Commun. 2020 Jun 29;11:3268. doi: 10.1038/s41467-020-17081-z (PMC7324362; doi:10.1038/s41467-020-17081-z)
Supplement: Supplementary file 10 — Supplementary Data 7 [file 41467_2020_17081_MOESM10_ESM.zip › File2/Confidence_High_Taxonomy/224522_out.krona.html]

Javascript must be enabled to view this page.

members
magnitude
magnitudeUnassigned
count
unassigned
taxon
rank

224522\_out

5

5
2
superkingdom

phylum
5
1239

class
5
186801

5
186802
order

541000
5
family

4

SRS018623\_contig\_number\_21090SRS050998\_contig\_number\_15523SRS144362\_contig\_number\_41283SRS971275\_contig\_number\_31370
1898205
species

1952413

SRS015578\_contig\_number\_contig-100\_1103.184123
1
species
